# Determinants of the Utilization of Diversified Types of Professionals for Mental Health Reasons in a Montreal (Canadian) Catchment Area

**DOI:** 10.5539/gjhs.v4n3p13

**Published:** 2012-05-01

**Authors:** Marie-Josée Fleury, Guy Grenier, Jean-Marie Bamvita, Michel Perreault, Jean-Caron

**Affiliations:** 1 Douglas Hospital Research Centre, Quebec, Canada; 2 Department of Psychiatry, McGill University

**Keywords:** mental disorders, professional utilization, health service utilization, health determinants, Canada

## Abstract

The study was designed to identify factors associated with the diversity of professionals consulted by 212 individuals affected by at least one mental disorder in the past 12 months in a Montreal catchment area. For inclusion in the study, participants had to be aged 15 to 65 and reside in the study zone. A comprehensive set of variables were analyzed in accordance with the Andersen’s behavioural model of health service use. General practitioners, psychiatrists, and psychologists were the main professionals consulted in this study. Having post-secondary education, more than a single mental disorder, excellent relationships with neighbours, and (marginally) being a lifelong victim of violence were associated with higher numbers of professionals consulted. As this study highlights the large number of diversified professionals consulted for reason of mental disorders, shared care initiatives may prove beneficial. Greater effort could also be made in increasing services toward those deemed more vulnerable.

## 1. Introduction

According to the World Health Organization, mental disorders represent 13% of the disease burden (WHO, 2011) and they also rate among the most common debilitating chronic diseases (Dhingra et al., 2010). A study covering 14 countries estimated the annual prevalence of mental disorders at 4.3% to 26.4% (Demyttenaere et al., 2004). This burden has prompted efforts to improve mental healthcare systems by strengthening community-based services and primary care. General practitioners are the healthcare professionals most commonly consulted by individuals with a mental disorder (Wang et al., 2006). They are considered less stigmatizing, more accessible, and are often more greatly appreciated than mental healthcare specialists (Zeiss & Karlin, 2008). Furthermore, as mental disorders are often closely associated with other chronic health problems, general practitioners can offer both physical and mental healthcare (Barr, 2000). In addition, treatment by primary care is three times less expensive than treatment by psychiatrists for comparable cases (Sturm & Klap, 1999).

The major role played by psychologists in treating patients with a mental disorder was also abundantly reported (Edlund et al., 2002; Howard et al., 1996; Lefebvre et al., 1998; Parslow & Jorm, 2000; Wang et al., 2003a), and in Quebec, they are the professionals most often consulted after general practitioners (Lesage et al., 2006). Psychotherapy and psychosocial treatments, particularly cognitive behavioural treatment (CBT) and Integrated Psychological Treatment (IPT), are potentially effective on their own or in combination with medication. Furthermore, up to 66% of individuals with depression prefer psychotherapy to medication (Mohr et al., 2010). Psychiatrists and other mental healthcare specialists however continue to occupy a central position in the treatment of mental disorders (Wang et al., 2006). A large percentage of individuals with severe mental disorders are supported both by a psychiatrist and case manager (generally a nurse or social worker) whose main function is to reduce hospital admission, increase use of community-based services, and enhance patient quality of life (D’Ercole et al., 1997).

Several epidemiological studies have investigated patterns of mental healthcare service use (Carr et al., 2003; Narrow et al., 2000; Parslow & Jorm, 2000; Vasiliadis et al., 2005; Wang et al., 2006). The most frequently used tool for identifying factors associated with service use is the Andersen’s behavioural model of health service use (Andersen, 1995). The model emphasizes the influence of individual and contextual dimensions on healthcare service use (Chartier-Otis et al., 2010). The behavioural model of health service use classifies predictors of service use into three categories: (a) predisposing, (b) enabling, and (c) needs-related factors. *Predisposing factors* are individual characteristics that exist prior to the illness such as age and gender. *Enabling factors* are features that influence care delivery and attitudes toward care and encompass variables such as income, social support, and geographical location. Finally, *needs factors* include assessments of physical and mental health by patients and professionals, including diagnoses and disorder severity.

Among predisposing factors, age, gender, marital status, education, race/ethnicity, territory, and country of birth are key determinants of professional consultations for individuals with a mental disorder. Several studies have found that individuals aged 25 to 44 (Leaf et al., 1985; Narrow et al., 2000), females (Parslow & Jorm, 2000) (Carr et al., 2003; Leaf et al., 1985; Narrow et al., 2000; Uebelacker et al., 2006; Vasiliadis et al., 2005; Wang et al., 2005b; Wells et al., 1986), previously married (Bebbington et al., 2000; Parslow & Jorm, 2000; Uebelacker et al., 2006; Wang et al., 2005b; Wells et al., 1986), better educated (Leaf et al., 1985; Parslow & Jorm, 2000; Vasiliadis et al., 2007), who are caucasian (Keyes et al., 2008; Leaf et al., 1985; Narrow et al., 2000; Wang et al., 2005b), and are non-immigrants (Kirmayer et al., 2007; Whitley et al., 2006) are most likely to consult professionals. Some predisposing factors also influence the types of professionals consulted. Individuals under 29 consult significantly more psychiatrists whilst older individuals seem to consult general practitioners (Carr et al., 2003; Wang et al., 2006). Females consult general practitioners significantly more (Carr et al., 2003; Wells et al., 1986) whereas males consult both psychiatrists and other mental healthcare specialists (Narrow et al., 2000; Shapiro et al., 1984; Wang et al., 2005b). Previously married individuals make greater use of general practitioners (Bebbington et al., 2000), while singles mainly consult psychologists (Wang et al., 2006). Finally, individuals with more limited education seem to consult both the clergy or other non-healthcare professionals whilst individuals with advanced education seem to consult significantly both psychiatrists and psychologists (Wang et al., 2003b).

Among enabling factors, perceived obstacles to care are negatively associated with service use (Leaf et al., 1985). Individuals in disadvantaged urban neighbourhoods and remote areas consult significantly less often (Meadows et al., 2002). Receipt of disability compensation is strongly associated with healthcare service use in general, specialized care, and (for those with no severe mental disorder) general practitioners (Narrow et al., 2000). Higher socio-economic status increases service utilization, especially psychiatrists or psychologists, even if individuals share the same private insurance coverage (Alegria et al., 2000; Hendryx & Ahern, 2001; Vasiliadis et al., 2007; Wang et al., 2000). However, social welfare and family allocations are associated with a greater use of specialized services among those with no severe mental disorder (Narrow et al., 2000). Family and social supports can be positively or negatively associated with service use (Albert et al., 1998; Carr et al., 2003; Pescosolido et al., 1998b). Professional support also plays a role (Bonin et al., 2007; Lemming & Calsyn, 2004). Having a regular source of medical care is positively associated with service utilization (Leaf et al., 1985).

Needs factors are most closely correlated to healthcare service use (Andersen, 1995). A diagnosis of schizophrenia, major depression or anxiety disorder (Leaf et al., 1985), co-morbid disorder (Lefebvre et al., 1998), duration of mental disorders (Lefebvre et al., 1998), psychological distress (Dhingra et al., 2010; Parslow & Jorm, 2000), poor physical health (Lemming & Calsyn, 2004) and maternal history of mental illness (Mojtabai et al., 2002) are needs-related factors associated with greater healthcare service utilization. By contrast, individuals with only substance abuse disorders use significantly fewer healthcare services (Andrews et al., 2001b). The nature of mental disorders also influences the choice of professional (Mojtabai et al., 2002). Individuals with panic disorder see significantly more psychiatrists and psychologists (Katerndahl & Realini, 1997). Individuals with depression are seen mainly by general practitioners (Howard et al., 1996). Individuals with substance abuse disorders are treated mainly by professionals other than psychiatrists, or a combination of psychiatrists and specialists (Howard et al., 1996). Finally, there is a significant link between the severity of the disorder and consulting psychiatrists exclusively (Wang et al., 2006).

Few studies have compared predictors of service use while taking into account concurrent consultations of one or more types of professionals. In 2006, Wang and colleagues identified six exclusive patterns of mental healthcare service use: (1) psychiatrist only; (2); general practitioner only; (3) general practitioner and other professionals; (4) other mental health specialists only; (5) human services only (defined as sector without psychiatrist, other mental health specialists or general practitioners), and (6) alternative resources only (Wang et al., 2006). By contrast, Meadows and colleagues consider that most individuals who see psychiatrists also consult other professionals and that many individuals consult general practitioners exclusively (Meadows et al., 1997). As shared care (close collaboration among psychiatrists, general practitioners, and psychosocial mental health professionals) is a leading trend in current healthcare reforms (Craven & Bland, 2006; Fleury et al., 2008; Fleury et al., 2009; Kates, 2002), a better understanding of variables that distinguish between single and multiple consultations is desirable.

Furthermore, several variables such as religious beliefs, neighbourhood/geospatial information, involvement in the justice system, impulsivity, and lifetime of aggressive behavior and violence, have received little or no attention in the literature to our knowledge. Some studies have found that churches were a significant factor of social integration in certain communities, and that church involvement was negatively associated with depressive symptoms (Latkin & Curry, 2003; Stockdale et al., 2007). Concerning neighbourhood/geographical variables, living in socio-economically deprived areas or socially disorganised neighbourhoods was associated with psychosocial stress and higher incidence of depressive symptoms (Galea et al., 2005). Finally, past or present experiences of violence, crime or imprisonment were all associated with higher prevalence of mental disorders (Stockdale et al., 2007). Those variables that were positively or negatively associated with the prevalence of mental disorders could also play a role in consultations of the diverse type of professionals.

The current study identifies factors associated with the diversity of professionals consulted by individuals diagnosed with a mental disorder in a 12-month period in a Montreal (Canadian) catchment area. A comprehensive set of variables were analyzed, based on the Andersen’s behavioural model (Andersen, 1995), which posits that health service use is determined by predisposing, enabling and needs-related factors.

## 2. Methods

### 2.1 Study Design and Setting

The study focused on an epidemiologic catchment area in Montreal, Canada’s second-largest city with a population of 3.6 million. The catchment area has a population of 269,720 and encompasses a wide variety of social structures, socio-economic status, education, healthcare service availability, and neighbourhood dynamics and security.

The catchment area included six neighbourhoods, ranging in population from 29,205 to 72,420. The percentage of immigrants is 25%, versus 26% in Montreal. Thirty-three percent of households (versus 23% in Quebec and 35% in Montreal) are low-income. Two of the six neighbourhoods encompass nearly half of the low-income population. The prevalence of psychological distress in the low-income population is high (Caron et al., 2007a). Mental healthcare services are delivered mainly by three providers: two health and social service centres (created through the merger of a general hospital, community local service centres, and nursing homes) offer first- and second-line healthcare services; and a psychiatric hospital provides second- and third-line services. Sixteen mental healthcare community-based agencies offer numerous services (crisis centre, day centres, self-help groups, advocacy, housing, job integration) for individuals with mental disorders and their relatives. About 40 medical clinics and 40 or so private psychologists and pharmacies complete the mental healthcare offer in the area.

### 2.2 Selection Criteria and Survey Sample

For inclusion in the survey, participants had to be aged between 15 and 65 and reside in the study area. The objective was to obtain a representative sample of the targeted population, both geographically, i.e. recruiting participants from all areas of the territory, as well as in proportion to the population density and in terms of socio-econonic statues (SES), that is, representative of the educational attainment structure of the territory. A target sample of 3,708 addresses was selected for recruitment. Participants were first contacted by phone and 300 individuals were recruited; however, it was soon decided to proceed otherwise due to the low response rate compared to the number of potential participants who were randomly selected. In order to improve recruitment, the original selected addresses were extended to a range of 14 neighbouring addresses. This range of 14 potential addresses included the original address, the three addresses that were the closest on each side of the main address, and the seven addresses on the opposite side of the street. We used 3,408 addresses for door-to-door recruitment; the 3,408 original addresses hence resulted in 3,408*14 neighbouring addresses, representing theoretically, a potential of 47,712 addresses where individuals would be available for recruitment. The recruiters met 7,265 individuals in persons and from those, 3726 (51%) refused to participate. Amongst the 3,539 individuals who accepted to participate, 1,405 (19%) were not eligible; 2,134 participants completed the interview for a cooperation rate at 49%. The response rate is superior to the median rates reported in epidemiological studies of populations conducted after the years 2000, when there was a steady decline in participation rates over the past 30 years (Morton et al., 2006; Singer, 2006).

The sampling was equally distributed in the study area among the various neighbourhoods (Caron et al., 2007b). Data were collected randomly from June to December 2009 by specially trained interviewers. Only a single participant by household was selected using procedures and criteria from the *National Population Health Survey* (NPHS, 2003-2005). For the recrutement, the interviewers contacted the individuals who agreed to participate in the study by phone within the week they were recruited, in order to schedule a face-to-face meeting either at the participant’s home or in an office designated for that purpose at the psychiatric hospital; however, most interviews were proceded at home. The face-to-face interview was conducted once a consent form was signed, and lasted approximately 1:30 minutes to 3 hours depending if a mental disorder was detected or not. Interviews were conducted using portable computers. Participants were given the option to choose the language (English or French) of their interview. The research was approved by relevant ethics boards. Each participant had the choice of giving the research team permission to contact him if a possible mental health problem was detected and to inform him about mental health services at his disposition if he felt the need to use them. For those aged 15 to 17, parents had to give authorization before the interview.

A randomly selected sample of 2434 individuals took part of the survey. The final study sample was overrepresented by women (62%) compared to the reference population (52%); men under the age of 45 were underrepresented as women of the same age group were overrepresented. In order to obtain the precise prediction of prevalence of mental illness in the population, we weighted the data for sex and age and did the same with the follow-up data. The mean age was 40,72 (ET = 14,08) of whom 49% were men, 38% were single, 45% were married or in a relationship, 12% divorced or separated, and 71% had a post-high school diploma; 79% were employed in the last 12 months and 25% were immigrants. French was the primary language spoken by 54% of the respondents, followed by English as the primary language in a proportion of 22% and 81% were White. The average personal income was 31 192$ CAN (SD-33) and the average family income 59 056$ CAN (SD = 49 851$); 33% of the respondents were considered as having a low income and 67% had none or a very low income. Among the 2,434 individuals who took part in the survey, 406 had experienced at least one episode of mental health disorder in the 12 months pre-interview, and were selected for the analyses described below.

### 2.3 Variables, Measurement Instruments, and Analyses

Assessed variables and measurement instruments are displayed in Table 1. Many instruments were used to measure specific health and psychosocial parameters (see Table 2). Variables were categorized according to Andersen’s behavioural model.

**Table 1:** Variables assessed in the study

		**Variables**
**Predisposing factors**	Socio-demographic Variables^i^	Age
		Gender
		Marital status
		Household composition and size
		Education
		First language
		Country of birth
	Religious beliefs	Importance attributed to spirituality
		Frequency of participation in religious activities
	Health beliefs	Quality of life^ii^
		Self-perception of mental and physical health^i^
	Justice system^i^	History of imprisonment

**Enabling factors**	Economic factors^i^	Income (personal, household; main source)
	Territory	Neighbourhood^iii-ix^
		Neighbourhood characteristics^iii-ix^
	Social support^x^	
	Social stigma^xi^	
	Geospatial variables^i^	Walking distance to healthcare services
		Driving distance to healthcare services
		Proportion of homeownership
		Proportion of rental accommodations
		Proportion of individuals who moved a year ago
		Unemployment rate among the population aged 25 and up
		Active population aged 15 years and up
		Average family income after taxes
		Average household income after taxes
		Proportion of recent immigrants

**Needs**	Mental disorders (number)^xii--xv^	
	Lifelong victim of violence^i^	
	History of aggressive behaviour^xvi^	
	Psychological distress^vvii^	
	Impulsiveness^xviii^	
	Emotional problems^1^	

**Health service utilization**^1^	Services are provided in hospitals (including hospitalization), mental health centres, rehabilitation centres, private clinics, pharmacies, and in the voluntary sector (e.g. support groups, crisis-line services).	
	Professionals consulted included: psychologists, general practitioners, psychiatrists, case managers, toxicologists, nurses, social workers, psychotherapists, pharmacists, other health professionals.	

Note: Measuring instruments are indicated by superscript numbers

**Table 2:** Measurement instruments

		Name	Description
**Predisposing** **factors**	I	Canadian Community Health Survey (CCHS) 1.2 (Statistics Canada, 2001)^*^	Survey questionnaire for socio-demographic characteristics; Yes/No and multiple choice questions; Likert and non-Likert scale questions
	II	Satisfaction with Life Domains Scale (SLDS)* (Baker & Intagliata, 1982)	20 items; 7-point Likert scale questions; Cronbach alpha: 0.92

**Enabling factors**	III	Sense of Community Scale (SCS) (Perkins & Long, 2002)	8 items; Cronbach alpha: 0.74
	IV	Community Participation Scale (CPS) (Saegert & Winke, 2004)	6 items; Yes/No and 4-point Likert scale questions; Cronbach alpha: 0.73-0.89
	V	Resident Disempowerment Scale (RDS) (Nario-Redmond & Coulton, 2000)	3 items
	VI	Sense of Collective Efficacy (SCE) (Sampson et al., 2002)	Evaluate the effect of social and institutional mechanisms on individuals living in the neighbourhood; Cronbach alpha: 0.80-0.91
	VII	Neighbourhood Disorder Scale (NDS) (Nario-Redmond & Coulton, 2000)	9 items; Cronbach alpha: 0.84
	VIII	Physical Conditions of the Neighbourhood (PCN) (Perkins & Long, 2002)	7 items; Cronbach alpha: 0.87
	IX	Facility in Neighbourhood (FN) (Coulton et al., 1996)	13 items; 10-point Likert scale questions; Cronbach alpha: 0.40 to 0.90

	X	Social Provisions Scale (SPS)* (Cutrona, 1989)	24 items; 4 point-Likert scale questions; Cronbach alpha: 0.92
	XI	Devaluation-Discrimination Scale (DDS)(Tohen et al., 2000)	12 items; 6-point Likert scale questions; Cronbach alpha: 0.68-0.99

**Needs**	XII	Composite International Diagnostic Interview (CIDI)* (Andrade et al., 2000)	Screening of mental disorders; included the most frequent mental disorders (depression, bipolar disorder, mania, post-traumatic stress disorder, anxiety disorders: social phobia, agoraphobia, and panic disorder)
	XIII	Drug Abuse Screening Test (DAST)* (Skinner, 1982)	20 items; yes/no questions; Cronbach alpha: 0.74
	XIV	Alcohol Use Disorders Identification Test (AUDIT)* (Bohn et al., 1995)	10 items; 2 or multiple choice questions; Cronbach alpha: 0.88
	XV	Parental Psychiatric History (PPH)(Driessen et al., 1998)	Measures mental disorders in parents and relatives; yes/no questions
	XVI	Modified Observed Aggression Scale (MOAS) for aggressive behaviours (Kay et al., 1988)	Assess 4 categories of aggressive behaviour: verbal aggression, aggression to propriety, self-inflicted aggression, physical aggression
	XVII	K-10 psychological distress scale (K-10 PDS)* (Kessler et al., 2003)	10 items; 5-point Likert scale questions; area under the receiver operating characteristic curve of SMI: 0.854
	XVIII	Barratt Impulsivity Scale (BIS)* (Barratt, 1985)	30 items; 4-point Likert scale questions

Univariate, bivariate and multivariate analyses were conducted. Univariate analyses were used to describe the sample as regards predisposing, enabling, and needs-related factors. Frequency distribution was calculated for categorical variables. Mean values and standard deviations were generated for continuous variables. Bivariate linear regression analyses encompassed a *t* test statistic between independent variables and the continuous dependent variable “number of professionals consulted,” with the Alpha value set at P < 0.10. Variables that yielded a significant association in bivariate analyses were introduced in the multiple linear regression model via the backward elimination technique (Alpha value set at P < 0.05). The model goodness-of-fit was estimated, as was the proportion of variance explained (R square). Variables included in the final model were used to compare participants who consulted four professionals and those who saw fewer than four professionals (categorical dependent variable). The cut-off value was applied on the basis of the mean value of the continuous dependent variable: the statistical distribution of the number of professionals used yielded a minimum of 1, a maximum of 12, and a mean of 3. For each comparison based on this categorical variable, a Chi-square P value was generated.

## 3. Results

Among the 406 individuals that have experienced at least one episode of mental health disorder in the 12 months prior the interview, was 56% female. The mean age was 39 (SD: 13.1). Eighty-one percent were Canadian-born. Mental and physical health, respectively, were deemed to be very good or excellent by 24% and 27% of participants. Approximately half (51%) reported they were satisfied or very satisfied with their lives. Almost half was employed (45%). Only 13% reported receiving social welfare. The mean household income was $43,650 (SD: $38,179; Minimum: 0; Maximum: 228,000.00). The mean score for quality of life was 94.7 (SD: 18.2; Minimum: 26; Maximum: 131). The three mental health disorders most frequently reported were: major depressive episode (52%); alcohol dependence (24%); and social phobia (20%). More than half (57%) of the participants reported having experienced emotional problems in the 12 months before the interview. Forty-nine percent declared being lifetime victims of violence, and 47% had a history of aggressive behaviour.

Among this 406 individuals, 212 (52%) reported at least a single episode of healthcare service use. Of the 212 participants, 63% were female (n = 134). Seventy-one percent completed post-secondary school education (n = 151). Eighty-three percent were single (n = 175). For 47% of the participants (n = 99), the main source of income was paid employment, while 12% (n = 25) received social welfare. The proportion of recent immigrants (less than one year) ranged from 3% to 26%, with a mean of 14% (SD = 7%). Many items assessing the quality of relationships with neighbours yielded scores ranging from 5 to 45, with a mean of 15 (SD = 9).

Among the 212 participants, the most commonly recorded mental healthcare disorders in the 12 previous months were: major depression (61%), alcohol dependence (20%), social phobia (19%), drug dependence (16%), and mania (11%), panic disorder (10%), and agoraphobia (9%). Seventy-six percent reported emotional problems in the 12 months prior the interview. The majority of the 212 participants were lifelong victims of violence (63%) or exhibited lifelong aggressive behaviour (55%). Among those who reported being lifelong victim of violence, 30% reported a history of unwanted sexual touching, 19% being beaten while being a child, 18% sexually assaulted, 18% beaten by sex partner or husband, 12% beaten by another person than sex partner or husband, 15% victim of theft or threatened with a firearm, and 9% physically abused.

Professionals consulted are displayed in Table 3. Most participants consulted general practitioners (63%), psychiatrists (58%) and psychologists (32%). These types of professionals were consulted more frequently by females than males; however, no statistically significant difference was found based on gender. Sixty-three percent of participants who sought help from psychologists had private health insurance. They are mainly female or individuals with higher household income (however, no statistically significant difference was found based on gender or income). As to participants’ age, the oldest were more likely to consult spiritual counsellors, while the youngest went to psychologists, psychotherapists, and social workers. Participants with post-secondary education tended to prefer services from pharmacists, educators, and psychotherapists and avoided spiritual counsellors. Participants who reported the highest household income favoured general practitioners, psychologists, and psychotherapists. Those who reported the lowest household income tended to see educators, nurses, and spiritual counsellors. Seventy-four participants (35%) consult both a general practitioner and a psychiatrist; forty-five (21%) consult both a psychologist and a general practitioner; thirty-five (17%) consult both a psychologist and a psychiatrist; twenty-eight (13%) consult all three types of professionals, and finally, forty participants (19%) consult four professionals or more. Participants who consulted more than four professionals were mostly females (55%), 40.7 years old on average (SD: 10.2), with a post-secondary diploma (80%), and a mean household income of $37,000.

**Table 3:** Main characteristics of participants as regards professionals consulted (n = 212)

		Gender				Age		Post-secondary education		Total household income	

		Males		Females							

	Total	n	%	n	%	Mean	SD	n	%	Mean $CAN	SD
Psychologist											
All	68	23	33.6	45	66.4	39.2	11.7	51	75.7	40844.8	40452.7
With insurance covering psychologist services	39	10	25.3	29	74.7	40.7	11.8	31	78.7	45513.0	43506.8
General practitioner (GP)	134	44	33.0	90	67.0	42.6	11.8	103	76.7	43036.5	38123.0
Psychiatrist	122	46	37.9	76	62.1	42.0	12.3	87	71.1	33519.9	34110.3
Social worker	43	19	43.7	24	56.3	39.2	12.1	34	77.6	27453.5	25222.2
Drug addiction counsellor	15	9	59.7	6	40.3	40.1	10.9	8	54.4	33251.8	27985.7
Psychotherapist	27	14	52.4	13	47.6	39.2	9.7	23	84.8	40416.7	31535.0
Nurse	20	11	53.5	9	46.5	42.4	11.2	15	75.7	20844.4	16408.7
Pharmacist	16	10	64.9	6	35.1	42.1	10.4	15	91.9	32432.7	42002.6
Educator	8	4	43.3	5	56.7	36.4	11.3	7	85.6	19615.6	14583.9
Spiritual counsellor	10	3	31.5	7	68.5	44.7	15.4	6	58.8	21418.3	20370.4
GP& Psychiatrist	74	27	35.8	48	64.2	43.5	11.5	57	76.4	39130.9	37797.2
Psychologist & GP	45	14	31.9	31	68.1	41.6	11.6	35	78.1	41151.5	36928.6
Psychologist & Psychiatrist	35	14	41.5	20	58.5	42.2	10.5	26	73.9	37164.1	37637.0
Psychiatrist & Psychologist & GP	28	14	49.4	14	50.6	42.6	11.1	21	75.7	38000.3	40627.8
4 professionals consulted or more	40	18	45.1	22	54.9	40.7	10.2	32	80.3	37804.9	37053.4

Table 4 displays professionals consulted according to diagnosis. The majority of participants with major depression, mania, dependence, anxiety or any combination of these disorders, sought help mainly from general practitioners, psychiatrists, and psychologists. General practitioners mainly served individuals with major depression and anxiety (78%) or only major depression (71%), and less often dependence (55%). Psychologists handled mania cases (44%) and less often dependence associated with mental disorders (31%). In cases where insurance covered the services of a psychologist, individuals who consulted psychologists especially had both major depression and anxiety. Psychiatrists managed all types of mental health disorders equally. Participants with mania were most likely (43%) to consult more than four professionals, including social workers, drug addiction counsellors, nurses, pharmacists, and spiritual counsellors.

**Table 4:** Frequency distribution of participants with mental health disorders in the twelve months before the interview according to professionals consulted (n = 212)

	Major Depression n (%)	Mania n (%)	Depen-dence: Alcohol or drug n (%)	Anxiety (panic disorder or agora-phobia or social phobia) n (%)	Major depression & anxiety n (%)	Mood disorders (major depression or mania) n (%)	Mood disorders & anxiety n (%)	Dependence & other mental disorders (depression or mania or anxiety) n (%)
Total Psychologist	129 (60.8)	23 (11.0)	47 (22.0)	67 (31.7)	36 (16.8)	136 (64.3)	165 (77.9)	26 (12.3)
All	43 (33.3)	10 (44.0)	15 (32.2)	26 (38.9)	13 (37.4)	46 (33.6)	57 (34.5)	8 (31.4)
With insurance covering psychologist services	28 (21.7)	5 (21.7)	11 (23.4)	16 (23.9)	10 (27.8)	29 (21.3)	34 (20.6)	6 (23.1)
General practitioner (GP)	92 (71.2)	16 (67.3)	26 (54.8)	45 (66.6)	28 (77.6)	95 (69.9)	111 (67.1)	17 (65.6)
Psychiatrist	78 (60.7)	15 (64.3)	29 (61.5)	40 (59.5)	23 (64.8)	83 (60.9)	98 (59.5)	17 (64.5)
Drug addiction counsellor	7 (5.5)	6 (25.4)	5 (10.5)	3 (4.6)	2 (4.4)	9 (6.5)	9 (5.6)	2 (8.8)
Nurse	14 (10.5)	5 (22.6)	7 (15.1)	5 (7.2)	3 (8.5)	14 (10.4)	16 (9.6)	4 (16.3)
Psychotherapist	17 (13.1)	3 (11.8)	6 (12.9)	9 (13.7)	4 (12.3)	18 (12.9)	22 (13.6)	2 (7.8)
Social worker	27 (21.0)	8 (34.3)	8 (17.8)	18 (26.1)	10 (26.8)	28 (20.7)	36 (21.9)	4 (17.2)
Educator	2 (1.3)	1 (2.4)	1 (2.5)	5 (7.0)	2 (4.8)	2 (1.7)	5 (3.2)	0 (0.0)
Pharmacist	11 (8.3)	5 (20.6)	5 (10.4)	5 (7.7)	3 (7.5)	11 (8.3)	14 (8.4)	4 (14.2)
Spiritual counsellor	8 (6.0)	3 (14.9)	3 (6.5)	6 (9.2)	5 (13.5)	8 (6.0)	10 (5.8)	2 (8.7)
GP& Psychiatrist	52 (40.7)	11 (46.2)	18 (39.5)	23 (34.3)	16 (45.8)	56 (41.1)	61 (37.0)	11 (43.7)
Psychologist & GP	32 (24.7)	7 (30.8)	9 (19.4)	19 (28.0)	11 (30.8)	32 (23.7)	40 (24.1)	6 (22.4)
Psychologist & Psychiatrist	26 (19.8)	7 (30.4)	9 (20.0)	13 (19.0)	8 (22.6)	27 (20.0)	31 (19.1)	6 (23.4)
Psychiatrist & Psychologist & GP	21 (16.3)	5 (22.2)	9 (18.5)	10 (14.5)	6 (17.3)	21 (15.7)	25 (14.8)	5 (20.8)
4 professionals consulted or more	28 (22.0)	10 (42.9)	7 (15.1)	15 (23.0)	10 (26.8)	30 (21.7)	35 (21.5)	5 (18.4)
Mean number of professional consulted [Mean (SD)]	2.6 (2.0)	3.6 (2.3)	2.6 (2.3)	2.7 (2.1)	2.9 (2.1)	2.6 (1.9)	2.6 (2.0)	2.8 (2.1)

Table 5 is a combination of two different analyses: (1) a multiple linear regression of variables significantly associated with the dependent continuous variable in bivariate analyses; and (2) descriptive comparative analyses of variables included in the linear multiple model. To compare participants who consulted four professionals or more (high-intensity users) and participants who consulted fewer than four professionals (low-intensity users), the continuous dependent variable was dichotomized as outlined in the “Analyses” section above. High-intensity users tended to have better relationships with neighbours, a greater number of mental health disorders, a history of aggressive behaviour (marginally significant), and post-secondary education. A greater proportion of recent immigrants in participating neighbourhoods were associated with the use of fewer than four professionals.

**Table 5:** Variables associated with the number of professionals consulted in the twelve months before the interview

	Variables significantly associated with the number of professionals consulted in the twelve months before interview in bivariate analyses	Comparison analyses: high users versus low users of professional services					Multiple linear regression; dependent variable: number of professionals consulted in the twelve months before interview			
		4 professionals consulted or more (n = 40)		Fewer than 4 professionals consulted (n=172)		P value (Chi-square)	95.0% CI			
							Beta	Sig.	Inferior limit	Superior limit

Predisposing factors	Post-secondary education [n (%)]	27	67.3	89	51.7	0.071	.205	.002	.072	.330
	Relationships with neighbours [Mean (SD)]	16.8	9.7	14.3	9.3	<0.001	.153	.023	.004	.059
Enabling factors	Proportion of recent immigrants in the neighbourhood (<1 year) [Mean (SD)]	12.3	7.4	14.2	7.2	0.008				
Needs factors	Number of mental health disorders in the 12 months before interview [Mean (SD)]*	5.9	1.9	1.7	.8	<0.001	.175	.010	.106	.766
	Lifelong victim of violence [n (%)]	28	69.4	105	61.1	0.352	.127	.061	-.025	1.063
	Lifelong aggressive behaviour [n (%)]	29	72.4	88	50.9	0.015				

Total variance explained: R square: 10.4%; F = 5.853; P < 0.001

The last four columns of Table 5 present the results for variables independently associated with the continuous dependent variable, “number of professionals consulted”. The model contains four variables, all positively associated. Having post-secondary education, a higher number of mental health disorders, and excellent relationships with neighbours and (marginally) being a lifelong victim of violence were associated with a greater number of professionals consulted.

## 4. Discussion

The study assessed variables associated with the number of diversified professionals consulted by individuals diagnosed with a mental disorder in a 12-month period in a 6-neighbourhood sector of Montreal, Canada. Using the Andersen’s behavioural model as a guide, a comprehensive set of variables was considered, including four categories of factors: predisposing, enabling, needs-related (independent variables), and the number of different professionals consulted (dependent variable).

Our results revealed that half the participants with mental disorders consulted one or more professionals for their condition. According studies, between 33% and 40% of individuals with mental disorders seek mental healthcare services (Andrews et al., 2001a; Kessler et al., 2005). According to the 2002 Canadian Community Health Survey of Mental Health and Well-Being (CCHS 1.2), 39% of Canadians use mental healthcare services (Lesage et al., 2006). The proximity of a psychiatric hospital may account for the overall greater healthcare service use and fewer visits to psychologists recorded in our catchment area. Compared to the Quebec population for which psychologists are the second most frequently consulted group (Lesage et al., 2006), psychologists were underutilized in our study. Individuals with low income have easy access to the public healthcare system (general practitioners and medication), but not to psychologists (where private insurance is mostly needed). A recent study has shown that cost is the main obstacle to psychotherapy access, especially for individuals with anxiety disorders (Chartier-Otis et al., 2010).

Our sample presents a very high proportion of individuals who have experienced lifelong violence or lifelong aggressive behavior. It could be explained by the fact that two of the four neighborhoods, low income and psychological distress are also particularly high. Individuals, particularly women which are the majorities in our sample, living in disadvantaged neighborhoods have more probability to rub criminals or violent individuals, and them be subject to experience violence (Rhodes et al., 2009; Stockdale et al., 2007). Furthermore, questions concerning lifelong victims of violence or lifelong aggressive behavior were purely subjective, participants having to answer yes or no. It is then impossible to precise when the violence act was committed, if it was a lonely act or not, and if the participants have conserved trauma.

Our results suggest that individuals who seek help for mental disorders generally consult several types of professionals. The follow-up of mental disorders in the community by general practitioners and other primary care professionals has been facilitated by the development of new antidepressant and other psychotropic medications with few side effects (Wang et al., 2006). Moreover, mental health reforms encourage increasing use of primary care and community-based services, which are considered less stigmatising, more accessible, and no costlier than hospital-based treatment (Thornicroft & Tansella, 2002). The use of diverse and increasingly community-based professionals is perceived as a positive development by various authors. Individuals who receive dual-modality treatment (for example, psychopharmacology and psychotherapy) are less likely to abandon treatment than individuals who consult psychiatrists only (Edlund et al., 2002). Furthermore, there is evidence that combining pharmacotherapy and psychotherapy is beneficial, especially in the treatment of major depression (Wang et al., 2006). In some countries, for example, England and Australia, models of care have been developed to improve access to psychotherapy for individuals with depressive or anxious disorder and integrate psychological treatment into general practice settings (Kyrios et al., 2010; Layard 2006).

Needs-related factors are generally considered as the prime predictors of healthcare service use. In our model, two variables (of four) associated with the greatest number of professionals seen for mental disorders are needs-related factors: the number of mental disorders and, marginally, being a lifelong victim of violence. Some studies have revealed that individuals who are treated both by general practitioners and mental health professionals have more severe mental disorders than individuals who consult general practitioners or mental health specialists only (Steel et al., 2006). Individuals suffering from multiple mental disorders have a higher level of distress and lower quality of life (Castel et al., 2006). They also present an increased risk of suicide, justifying the use of specialized services (Merikangas et al., 1998). Due to their greater psychological suffering, individuals with multiple mental disorders may be more motivated to seek help (Castel et al., 2006; Kessler et al., 1999).

The same explanations may be applied to individuals who are lifelong victims of violence. Several studies reveal that exposure to violent experiences increases the risk of developing a mental disorder (Hedtke et al., 2008; Rhodes et al., 2009; Stockdale et al., 2007; Teplin et al., 2010). Females who were victims of physical and sexual violence are up to 14 times more likely to develop post-traumatic stress disorder and five to eight times more likely to experience a major depressive episode than those who experience no violence (Hedtke et al., 2008).

Several studies have shown that individuals with higher education are more likely to use mental healthcare services (Leaf et al., 1985; Vasiliadis et al., 2005; Vasiliadis et al., 2007). According to Howard and colleagues (Howard et al., 1996), individuals with the highest level of education are the less least likely to develop a mental disorder but the most likely to consult mental healthcare professionals. This apparent paradox may be explained by the fact that individuals with higher education generally have greater financial resources to pay for private services such as psychologists (Barr, 2000; Wang et al., 2005b). It may also reflect their greater propensity to acknowledge their condition and greater receptivity to mental healthcare professionals (Olfson et al., 2002; Young et al., 2001). Lack of awareness concerning the nature of mental disorders and lower receptivity to mental health professionals may hinder access to treatment for individuals with less education and lower socio-economic status (Howard et al., 1996; Leaf et al., 1985; Young et al., 2001). Individuals with higher education may be more hard to please and more likely to negotiate healthcare services.

The association between higher numbers of professionals consulted and better relationships with neighbours may seem unexpected. Several studies have linked mental health and social disorder (Latkin & Curry, 2003; Ross & Mirowsky, 2001). Neighbourhood stressors are associated with depression, anxiety, substance abuse, and psychological distress (Stockdale et al., 2007). Furthermore, social support helps to protect against mental disorders (Pirkola et al., 2005), and healthcare service use increases when social networks slacken (Albert et al., 1998). Lack of social support is associated with increased hospital admission (Albert et al., 1998; Becker et al., 1997; Guzzetta et al., 2010). However, social networks can help individuals to recognize their problems and encourage them to seek professional help (Albert et al., 1998; Howard et al., 1996; Pescosolido et al., 1998a). According to Pescosolido’s network episode model (Pescolido, 1992), individuals do not always make health-related decisions alone (Bonin et al., 2007). A larger social network would be associated with greater coercion from family and friends to seek professional help (Pescosolido et al., 1998a; Steel et al., 2006). As reported in previous studies, many individuals do not seek treatment for their mental disorders; when they do, they generally see various professionals, and close to 20% consulted at least four mental health-care professionals.

### 4.1. Limitations

The study includes some limitations. First, the high proportion of experienced lifelong violence could impact the generalizability of the results. Second, the severity of mental disorders was not considered. Previous studies have reported that severe cases use more services than mildly severe or moderate ones (Tempier et al., 2009; Wang et al., 2007). Third, there was no information on the frequency of utilization. It is possible than individuals who consult only one professional tend to consult him or her more often. Finally, our data cannot adequately address important areas as waiting times or quality of care which play a crucial role in service use (Wang et al., 2005a).

## 5. Conclusions

The present study was the first study to highlight the large number of diversified professionals consulted for reason of mental disorders – to our knowledge. It was also of interest as it analysed a comprehensive set of variables, including geospatial factors. To our knowledge, no previous study has focused on such numerous variables using the Andersen behavioural model. To improve the efficiency of the mental healthcare system, a better understanding of factors associated with consultations of one or more professionals is needed. The study revealed the association between having more serious mental health disorders (number of mental health disorders and marginally, being a lifelong victim of violence) and the number of professionals consulted. The study also demonstrates that positive relationships with neighbours and higher individual education enabled increased professional consultations. Socioeconomic statues may play a defining role both in type of neighborhoods and higher education level. Greater effort should be made to increase services toward the more vulnerable individuals, who can be both stigmatized because their mental disorders. Furthermore, shared-care initiatives, thereby favouring improved coordination among psychiatrists, general practitioners and other professionals, particularly psychologists, should be promoted as our study showed that most individuals consulted these professionals concurrently.
